# Sex/gender differences in general cognitive abilities: an investigation using the Leiter-3

**DOI:** 10.1007/s10339-024-01199-9

**Published:** 2024-05-15

**Authors:** David Giofrè, Enrico Toffalini, Lorenzo Esposito, Cesare Cornoldi

**Affiliations:** 1 Disfor, University of Genoa, Genoa, Italy; 2 Department of General Psychology, University of Padua, Padua, Italy

**Keywords:** Intelligence, Sex/gender, Leiter, MGFCA

## Abstract

Research has shown that differences between males and females are not in general intelligence, but only in some specific factors and tasks. We used the Italian standardization of the Leiter-3, which is a completely nonverbal cognitive battery, to investigate the nature of sex/gender (we used sex/gender to reflect the awareness that the effects of biological ‘sex’ and socially constructed ‘gender’ cannot be easily separated and that most individuals’ identities are informed by both sex and gender) differences. In doing so we used a multigroup confirmatory factor analysis approach. Results confirmed that males and females perform similarly in general intelligence, but present with some specificities. Males perform better on some, but not all, tasks requiring the spatial manipulation of the stimuli, but females consistently outperform males in tasks such as the nonverbal Stroop, requiring inhibition and attention control to a larger extent. The clinical and practical implications of our findings are considerable. The identification of specific cognitive strengths and weaknesses in males and females underscores the importance of tailored approaches in clinical assessments and interventions.

## Introduction

Differences between males and females in cognitive abilities have been repeatedly investigated. Though there has been some debate, several batteries have been used to evaluate differences in intelligence between males and females, with the Wechsler Intelligence Scale for Children (WISC) being the most widely used. Giofrè and co-authors (2022) conducted a meta-analysis of WISC batteries, revealing that there were no significant differences between males and females in general intelligence, while some differences endured on specific factors and subtests. For example, a difference favoring females was found in the coding subtest, which is a measure of the processing speed and requires writing symbols associated with other stimuli as soon as possible. At the same time, males seem to consistently perform better in some visuospatial tasks, such as the block design, which requires mentally manipulating the stimuli (Giofrè et al. 2022a, b). These differences are relatively stable and have been found in several other subgroups, including children with specific learning disabilities, attention-deficit/hyperactivity disorders, and autism spectrum disorders (Giofrè et al. 2023, 2024; Giofrè et al. 2022a, b). However, it is essential to consider that the specific battery used might influence these differences. Therefore, confirming these findings with alternative batteries is crucial. Indeed, a wealth of evidence suggests that distinct intelligence batteries may yield different results, and the presence of sex/gender (we used sex/gender to reflect the awareness that the effects of biological ‘sex’ and socially constructed ‘gender’ cannot be easily separated and that most individuals’ identities are informed by both sex and gender) differences could be closely tied to the chosen battery. This phenomenon may arise as a consequence of the construction of the IQ tests (Mackintosh 2011, p. 184).

It can be argued that an assessment of intelligence, predominantly reliant on language, may influence the evaluation of differences between males and females in intelligence. For example, the WISC-IV heavily relies on verbal instructions, features numerous verbal subtests, and assesses working memory only using alphanumerical materials. In this context, the Leiter-3 offers a valuable alternative, as it is a completely nonverbal intelligence battery, in which the examination is entirely nonverbal. The battery assesses three major dimensions of cognitive ability (Roid et al. 2013): nonverbal intelligence (IQ), measured by four main subtests; nonverbal memory, measured by two main subtests, and attention and interference control, measured by two main subtests. Despite encompassing three different factors, the battery follows a hierarchical approach, with a superordinate factor (i.e., the g-factor) at the top of the hierarchy (Carroll 1993). The rationale behind the development of Leiter-3 is to provide a nonverbal assessment tool suitable for participants with different disabilities, particularly those with limited verbal abilities (Roid et al. 2013).

The Leiter-3 stands apart from other intelligence scales due to its distinctive features. In this scale, a single nonverbal intelligence factor is assessed through a combination of visual (gV) and fluid intelligence (gF) tasks, offering a comprehensive measure of general intellectual functioning. The other subtests, however, do not concur with the calculation of the nonverbal IQ. This approach is based on the fact that the scale is recommended for children with atypical development, in which working memory and processing speed are frequently impaired (Cornoldi et al. 2014; Giofrè et al. 2017; Giofrè and Cornoldi 2015). For example, the direct comparison of this scale with the WISC-IV, shows that the Leiter-3 provides different estimates of intelligence in children with autism spectrum disorders (Giofrè, Provazza, et al. 2019a, b). An additional distinctive feature lies in the incorporation of an attention and interference control factor, designed to assess both processing speed and the ability to manage interference. This is accomplished through the inclusion of a nonverbal Stroop measure in the assessment, demanding both processing speed and interference control (Roid et al. 2013). Finally, unlike the WISC-IV, where the evaluation of working memory is entirely verbal, or the WISC-V, featuring a mix of verbal and nonverbal working memory tasks, the Leiter-3 exclusively employs nonverbal measures for assessing working memory. For all these reasons, the Leiter-3 holds the potential to offer important insights into the assessment of intellectual functioning, and into the possible presence of differences between males and females.

Using a large and representative sample of participants Hedges and Nowell (1995) found subtle yet consistent differences on certain tasks. For example, males were generally favored by tasks tapping on spatial relations, mechanical reasoning, and spatial abilities (Voyer et al. 1995), while females consistently exhibited superior performance in verbal fluency tasks as well as in speed-related tasks (e.g., Halpern and Wai 2019). On the other hand, differences in other areas remained elusive, e.g., studies typically fail to observe differences in verbal WM tasks (Giofrè et al. 2022a, b), and although differences in spatial working memory were detected, their magnitude was relatively small (Voyer et al. 2017).

Another problem with the aforementioned literature is that differences between sexes/genders are seldom examined using more advanced statistical techniques, such as a multi-group confirmatory factor analysis approach (MGCFA). Measurement invariance, assessed via MGCFA, is a statistical method that enables to estimate whether a test is measuring the same construct across different groups. When measurement invariance is confirmed, both observed and latent variables are expected to be invariant across groups. Several steps are taken for testing measurement invariance: configural invariance, where the same structure is imposed between groups; metric, which requires the imposition of identical loadings on the groups; scalar, which forces the intercepts to be equal. Additionally, more stringent forms of invariance, such as latent means, latent residuals, and latent covariances, can be assessed. Even when small differences exist across groups, more lenient forms of invariance, such as partial invariance, can be assessed, allowing some, but not all, parameters to be freely estimated between two groups.

The aim of this report is to evaluate the presence of differences between males and females at the Leiter-3. This capitalizes on a standardization study conducted on a large group of Italian participants. To achieve this aim, we employed a MGCFA, enabling us to estimate differences at the latent level. In accordance with existing literature, we did not expect to find differences in the g-factor. However, we anticipated observing some differences, favoring females in the attention, speed, and interference control subtests, since females outperform males in tasks tapping attentional control (Geary et al. 2021), and favoring males in some other subtests, due to the involvement of visuospatial abilities (Geary 1995).

## Methods

### Participants

We considered the Italian standardization sample of the Leiter-3 (Cornoldi et al. 2016), excluding children with various disabilities. The standardization process comprised two phases: an initial pilot phase involving a smaller sample of children subdivided across different geographical areas, followed by a second phase that involved a larger sample. The sample was stratified for age, sex, and other demographic variables, such as years of education, parents' education, profession, and geographical area, based on the latest ISTAT (Italian demographical institute) data. The sample included a proportion of children with some neurodevelopmental conditions (about 7%). The sample aimed to be representative of the Italian population of children and adults from 3 to over 60 years. The overall reliability of the scale was deemed satisfactory, with results aligning closely with those obtained in the original USA version. Confirmatory factor analyses confirmed the existence of three lower-order factors and of a g-factor at the top of the hierarchy. All loadings were high, generally exceeding 0.55, with particularly robust loadings on the g-factor, typically higher than 0.70.

In this paper, we included a sample of 540 participants, without any accompanying disability. The sample was stratified and included 13 age groups (from 3/4 years of age to > 60 years of age). As mentioned above, participants were matched for sex/gender and other demographic variables. The number of males and females was similar in the overall sample, females = 49%, *χ*^*2*^(1) = 1.067, *p* = 0.302, and in each age group, *χ*^*2*^(12) = 10.80, *p* = 0.546.

### Measures

The Leiter-3 includes three distinct nonverbal batteries: the IQ, the memory and the attention, speed, and interference control batteries (Cornoldi et al. 2016; Roid et al. 2013).

### Nonverbal intelligence battery (NVI)

The nonverbal IQ encompasses tasks assessing fluid intelligence components following the classical hierarchical approach (Carroll 1993).

*Figure Ground (FG)* requires the identification of embedded figures, or designs, within a complex stimulus. *Form Completion (FC)* tests the ability to recognize a "whole object" from a randomly displayed array of its fragmented parts. *Classification/Analogies (CA),* begins with tasks that measure categorization of objects or geometric designs, followed for older children by matrix analogies items using geometric shapes. *Sequential Order (SO)* consists of selecting logically-related visual stimuli that progress in a corresponding order.

### Nonverbal memory battery (NVM)

The nonverbal memory battery is constituted by two subtests.

*Forward Memory (FM)* tests the ability to recall a sequence of pictured objects test measures the capacity to remember the same sequence but in the opposite order as indicated by the examiner. In addition to recalling the sequence, individuals must inhibit the previous sequential information that may be stored. Both memory subtests gauge the span of immediate retention. They bear resemblance to the digit span subtest of the Wechsler tests, albeit presented in a nonverbal format. Both forward and backward memory tap into some common constructs, although they involve distinct mental abilities. Digits forward is associated with attention efficiency, while digits backward is linked to working memory, involving the transformation of information while in short-term memory storage (Cornoldi et al. 2013). This distinction is likely to hold true with the nonverbal presentation of stimuli. These subtests can measure attention, short-term memory, and working memory. In this context, executive functioning likely plays a more critical role in performance than mere intact memory.

### Attention and interference battery (NVAI)

Tasks included in this battery are tapping processing speed, attention, and interference control. These tasks are related to the concept of speed as formulated by Carroll (1993). However, this battery is based on a more refined neuropsychological approach, stressing attention and control of interference over speed per se.

*Attention Sustained (AS)* consists of "boring" clerical tasks such as finding, and crossing-out, all squares found in an array of geometric shapes printed on a page. The *Nonverbal Stroop (NS)* is a test designed to assess cognitive processes, neuropsychological deficits, and the control of interference. This task is explicitly designed to measure the ability to inhibit responses to distracting stimuli, thereby minimizing cognitive interference. Lower scores on the Nonverbal Stroop suggest that the individual faces challenges in overcoming cognitive interference related to physical marking and color discrimination (Roid et al. 2013).

### Statistical approach

Analyses were performed with R (R Core Team 2024), and using the *lavaan* package (Rosseel 2012). Our analytic strategy involved two steps. First, CFAs were performed to evaluate the factor structure and to choose the most suitable model. Then, the selected model was tested using MGCFA to test measurement invariance between males and females.

Different goodness-of-fit statistics were computed to evaluate model fit (Hu and Bentler 1999). In particular, the chi-square (*χ*^*2*^), the root mean square error of approximation (*RMSEA*), the standardized root mean square residual (*SRMR*), the comparative fit index (*CFI*), the non-normed fit index (*NNFI*), and the Akaike information criterion (*AIC*) were considered. Cut-off values were considered good if *CFI* and *NNFI* were greater than 0.95, *RMSEA* was lower than 0.06, and *SRMR* was lower than 0.05. The most plausible model was selected based on goodness-of-fit criteria, by considering the difference in relative indices (e.g., the AIC), between competitive models.

### Statistical analyses

After selecting the model, we tested measurement invariance across males and females using MGCFA. Absence of chi-square significance difference (*Δχ*^*2*^), and lower AIC values were considered for testing model invariance. To evaluate invariance between males and females, we also considered the general guidelines proposed by Chen (Chen 2007). A decrease of *CFI* less than 0.01 (*ΔCFI*), an increase of *RMSEA* less than 0.015 (*ΔRMSEA*) between models, and acceptable model fit indices are claiming for model invariance (Cheung and Rensvold 2002). Similarly, a decrease of *NNFI* lower than 0.01 (*ΔNNFI*) and an increase of SRMR lower than 0.015 (ΔSRMR) were considered acceptable for invariance.

We followed a series of steps to test measurement invariance. First, we tested configural invariance. In this step, all model parameters are free to vary across groups. If the fit indices are acceptable, the model configuration was regarded as adequate and configural invariance is established. Second, metric invariance was assessed by constraining factor loadings to equality for the two groups. If this model did not substantially lose fit, metric invariance is established. In the third step, scalar invariance was tested by constraining intercepts to equality across groups. Fourth, invariance of latent means was tested by constraining latent means to zero in both groups. Once all these steps were completed, strict invariance was established, and it implied that the two groups could be directly compared on their latent variable scores. The subsequent steps were testing the equality of variances and residuals of the latent factors. If at any step invariance was not reached, one parameter at a time was freed to check whether a partial invariance could be established.

## Results

Descriptive statistics, and standardized differences, for males and females, are presented in Table 1. Results show that there were overall small differences between the two groups with some tasks favoring males, while others favoring females. Looking at the main indices, there were trivial, albeit not statistically significant, differences favoring males in the nonverbal QI and nonverbal memory, while differences in attention, speed and control of interference were larger, statistically significant, and favoring females. As for the subtests, females presented with higher performances in the FG, while in the remaining tests tapped by the nonverbal IQ males had higher performances. The situation was mixed for tasks tapping nonverbal memory, with females outperforming males on FM, while the opposite was true for RM, these differences, however, were small in magnitude and not statistically significant. Finally, females presented higher scores at both ASC and NS, with differences being larger and statistically significant for NS.

**Table 1:** Mean standardized scores at the Leiter-3 battery obtained by females and males

	Females		Males		*d*
	M	SD	M	SD	
Figure Ground	11.16	2.79	10.59	3.11	− 0.19*
Figure Completion	9.93	2.60	10.39	2.74	0.17*
Classification/Analogies	10.08	2.66	10.61	2.67	0.20*
Sequential Order	10.31	2.76	10.65	2.82	0.12
Forward Memory	9.98	2.64	9.92	2.59	− 0.02
Reverse Memory	9.98	2.69	10.21	2.58	0.09
Attention Sustained	10.40	2.56	10.15	2.62	− 0.09
Nonverbal Stroop	10.49	2.50	9.84	2.66	− 0.25*
NVI	100.74	13.97	101.31	14.62	0.04
NVM	100.83	14.30	101.36	13.55	0.04
NVAI	103.00	13.42	100.24	13.90	− 0.20*

*d* = Cohen’s d (positive values favoring males)

*NVI* nonverbal intelligence, *NVM* nonverbal memory, *NVAI* nonverbal attention and interference

*p < .05

### Confirmatory factor analyses (CFA)

We tested several models to evaluate the structure of intelligence using the Leiter-3. We first tested a single factor structure, but the fit of this model (Model 1; Table 2) was not satisfactory. We then went on testing a hierarchical model with three factors (NVI, NVM, and NVAI) at the bottom, and a single g-factor (g) at the top of the hierarchy. This model provided a very good fit with the data (Model 2; Table 2). It is worth noting that loadings from the g-factor to the lower order factors were high (0.89, 0.76, and 0.68 for the NVI, NVM, and NVAI factors respectively). These findings seem to indicate that a superordinate g-factor with strong loadings on secondary factors can be found in the Leiter-3. It is worth mentioning, however, that it is impossible to compare this model with a simple three intercorrelated factor model, which has an identical fit, as it has the exact same degrees of freedom. We finally tested a bifactor model with three factors (NVI, NVM, and NVAI) and a g-factor (g) loading on each subtest. This model was less parsimonious as compared to the previous one (i.e., had less degrees for freedom; Table 2), but it was not statistically superior compared to the classical hierarchical model, *Δχ*^*2*^ = 0.563, *p* = 0.905, and had a higher AIC. We therefore decided to retain the classical hierarchical model of intelligence for subsequent analyses.

**Table 2:** Fit indices for the structure of the Leiter-3 in the overall group

	*χ*^*2*^	*df*	*p*	*RMSEA*	*SRMR*	*CFI*	*NNFI*	*AIC*
Model 1	130.13	20	.000	.101	.059	.862	.807	20,139
Model 2	30.49	17	.023	.038	.025	.983	.972	20,046
Model 3	29.93	14	.008	.046	.025	.980	.960	20,051

### MGCFA

We performed a series of progressively stricter MGCFA, testing configural, loadings, intercepts, latent means, residuals, and latent variances. When the full invariance was not established, we attempted, based on modification indices and on theoretical reasons, to free up some parameters to establish partial invariance.

In the first model (M1) the same structure was imposed on the two groups but allowing all other parameters to be freely estimated in the two groups. This model provided a good fit (Table 3). Therefore, we decided to go on with stricter forms of invariance. In this second model (M2), loadings were constrained to be equal in the two groups. Also in this case, the fit was good (Table 3), the model was not statistically different from the previous model, had a lower AIC, and fit indices did not change markedly (M2 vs. M1; Table 4). The invariance of the loadings was therefore established. In a third model (M3), we went on constraining the intercepts in the two groups (Table 3). To allow convergence we also fixed the latent mean of the g-factor to be invariant between the two groups. In this case, the fit was less favorable. The model had a higher AIC, the chi-square difference test was statistically significant (M3 vs. M2; Table 4), and fit indices were worse. This pattern suggested the presence of some differences in some intercepts. Looking at the parameters and at the individual intercepts we noticed that one intercept (i.e., the intercept of Figure Ground, was largely different in the two groups), we therefore went on testing a partial invariant model (M3b; Table 3), allowing the intercept of Figure Ground to be freely estimated in the two groups. This model (M3b) provided a good fit (Table 3), was not statistically different from the model in which only loadings were constrained (M3b vs. M2; Table 4), presented with an adequate fit, and was therefore retained.

**Table 3:** Fit indices for MGCFA models tested

Constraints	Model	*χ*^*2*^	*df*	*P*	*RMSEA*	*SRMR*	*CFI*	*NNFI*	AIC
Structure	M1	59.71	34	0.004	0.053	0.032	0.969	0.948	20,062
Loadings	M2	67.58	41	0.006	0.049	0.040	0.968	0.956	20,056
**Intercepts**	**M3**	**90.56**	**46**	**0.000**	**0.060**	**0.047**	**0.946**	**0.934**	**20,069**
Partial intercepts	M3b	71.98	45	0.006	0.047	0.042	0.967	0.959	20,053
**Latent means**	**M4**	**91.84**	**48**	**0.000**	**0.058**	**0.051**	**0.946**	**0.938**	**20,067**
Partial latent means	M4b	72.28	46	0.008	0.046	0.042	0.968	0.961	20,051
Residuals	M5	76.81	54	0.022	0.040	0.043	0.972	0.971	20,039
Latent variances	M6	81.65	58	0.022	0.039	0.049	0.971	0.972	20,036

Models with a worse fit are in bold

**Table 4:** Test of invariance between males and females

Model comparison	*Δχ*^*2*^	*Δdf*	*p*	*ΔAIC*	*ΔRMSEA*	*ΔSRMR*	*ΔCFI*	*ΔNNFI*
M2 vs. M1	7.87	7	0.345	− 6.13	− 0.004	0.008	− 0.001	0.007
**M3 vs. M2**	**22.97**	**5**	**0.000**	**12.97**	**0.011**	**0.007**	− **0.022**	− **0.022**
M3b vs. M2	4.40	4	0.355	− 3.60	− 0.002	− 0.006	0.021	0.025
**M4 vs. M3b**	**19.86**	**3**	**0.000**	**13.86**	**0.011**	**0.010**	− **0.021**	− **0.021**
M4b vs. M3b	0.30	1	0.583	− 1.70	− 0.001	− 0.009	0.021	0.023
M5 vs. M4b	4.53	8	0.807	− 11.47	− 0.006	0.001	0.004	0.010
M6 vs. M5	4.84	4	0.304	− 3.16	− 0.001	0.005	− 0.001	0.001

Statistically significant models with a worse fit are in bold

**Fig. 1:**
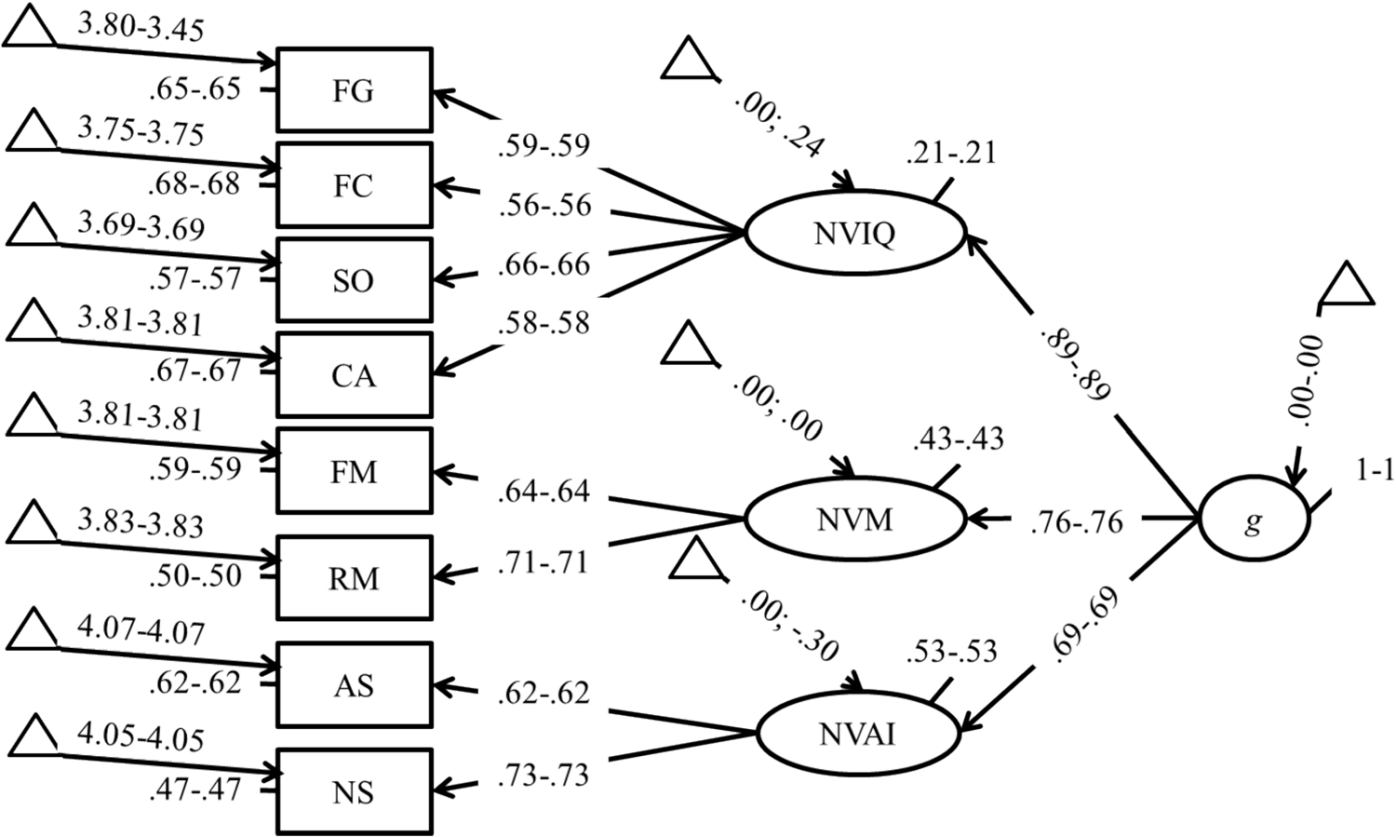
Path diagram of partial measurement invariance model (M6), females on the left and for males on the right. *FG* Figure Ground, *FC* Figure Completion, *CA* Classification/Analogies, *SO* Sequential Order, *FM* Forward Memory, *RM* Reverse Memory, *AS* Attention Sustained, *NS* Nonverbal Stroop, *NVIQ* Nonverbal IQ, *NVM* Nonverbal Memory, *NVAI* Nonverbal Attention and Interference

In a subsequent model we went on constraining latent means to be equal (M4; Table 3), in this case, the AIC was higher, the difference between this model and the previous one was statistically significant (M4 vs. M3b; Table 4), and the fit was not very satisfactory. Looking at the unconstrained model (i.e., Model 3b), we noticed in fact that some latent means were not invariant across the two groups (i.e., for the NVIW, and NVAI factors). In a further model (M4b; Table 3) we decided to free up the latent mean of NVIQ and of NVAI in the two groups. In this case, the fit improved considerably (Table 3), the AIC was lower, the model not statistically significant (M4b vs. M3b; Table 4), and all other parameters claimed in favor of model invariance (Table 4). It is worth noting that this model indicates the presence of a latent mean difference favoring females in the residual NVAI, and a difference favoring males in the residual NVIQ. It is also to note that in this model the intercept of Figure Ground, which was favoring females, was freely estimated, which makes it very hard to establish differences in this specific factor. We then went on constraining latent residuals (M5; Table 3) and latent variances (M6; Table 3). Also in this case, all parameters claimed in favor of model invariance of latent residuals (M5 vs. M4b; Table 4), and of latent variances (M6 vs. M5; Table 4). The final model (M6), in which the intercept of FG, the latent mean of NVIQ and NVAI were free to vary across groups, is presented in Fig. 1.

## Discussion

The main aim of this paper was to assess potential differences between males and females in the Leiter-3, an intelligence battery characterized by a completely nonverbal intelligence assessment. Our results confirm our main hypothesis: no statistically significant differences were observed in the g-factor. This finding aligns with a substantial body of research spanning various batteries, age groups, countries and, realities, in which differences in the g-factor, tend to be small and of trivial magnitude (Colom et al. 2000; Haier et al. 2005; Lubinski 2000; Mackintosh 2011). However, some residual differences did emerge in the lower order factors after accounting for the g-factor.

As for the structure of intelligence, the Leiter-3 does not facilitate the calculation of a g-factor loading on all main subtests. Such a decision is grounded in theoretical reasons. The battery is in fact commonly employed with children with disabilities, often facing impairments in working memory and processing speed (e.g., Cornoldi et al. 2014; Giofrè, Pastore, et al. 2019). However, examining the loadings of the g-factor on the second-order factors reveals a robust association between the g-factor and these second-order factors. This implies that a single g-factor can account for a significant and large portion of the variance in the lower-order factors. Furthermore, the measurement of intelligence in the Leiter-3, represented by the nonverbal intelligence IQ, relies on only four subtests. Our data indicate that incorporating all subtests, not just the four main ones, for calculating the IQ could be crucial, particularly for typical populations; and in fact working memory, attention, speed, and interference control are identified as significant determinants of intellectual functioning (e.g., Engle et al. 1999; Giofrè et al. 2013; Salthouse et al. 2008). Using all available information for IQ calculation could prove advantageous from both research and clinical standpoints. Comparing the scatter between an overall measure of intellectual functioning and a measure incorporating nonverbal memory, attention, and interference control could provide valuable clinical insights, particularly in assessing individuals with neuropsychological deficits (e.g., Giofrè et al. 2017).

The observed advantage of females over males in the attention, speed, and interference control within the Leiter-3 battery is particularly intriguing. It is noteworthy that the Leiter-3, in contrast to Wechsler batteries, replaced the processing speed factor with a factor that also incorporates attention and interference control. This decision aligns with existing evidence suggesting that attentional and interference control significantly contribute to intelligence (e.g., Shipstead et al. 2012). Our results highlight that, even though language is not directly implicated, females demonstrate superior performances in both speeded tasks, such as perceptual speed tasks (Hedges and Nowell 1995), and in the underlying factor, with differences more pronounced in the nonverbal Stroop (Geary et al. 2023). This finding aligns with a recent meta-analysis indicating a female advantage in inhibition control tasks (Sjoberg et al. 2023). It is also consistent with evidence demonstrating that females exhibit lower rates of ADHD and are generally considered more proficient in tasks requiring attentional control and inhibition (Geary et al. 2023).

As for the nonverbal IQ battery, the situation is very interesting. We found that one subtest, Figure Ground, which requires finding information in a complex background, was favoring females over males while the others were generally favoring males. This finding is in accordance with research indicating that in some spatial tasks, including spatial location and object location tasks, and in tasks requiring paying attention to the details, females typically tend to exhibit higher performances (Eals and Silverman 1994; Lange-Küttner and Ebersbach 2013; Tottenham et al. 2003). It is also worth mentioning that tasks included in the nonverbal intelligence battery are heterogeneous. Some tasks are measuring merely visual factors, while others require the mental rotation of the stimuli. In fact, males tend to perform better in tasks requiring to mentally rotate and bind scattered pieces (Johnson and Bouchard 2007; Voyer 2011; Voyer et al. 1995), which might explain the observed advantage we found in some spatial tasks. This is in accordance with a wealth of evidence indicating that males perform better in tasks requiring the mental manipulation and rotation of spatial stimuli (Geary 2022; Geary et al. 2021, 2023). It is also worth mentioning that this difference was found in the residual variance (see Johnson and Bouchard 2007 for the rationale), after accounting for all the variance related to the g-factor, which explains most of the overall variance. In summary, our findings suggest that the male advantage in spatial tasks is not universal, as it appears contingent on specific factors and tasks withing the nonverbal IQ battery.

The current paper supports previous findings using MGCFA. Studies comparing males and females with different intelligence batteries and employing MGCFA have identified no differences in the second-order g-factor, and only minimal differences in some other factors, including a difference favoring males in the perceptual organization factor of the WAIS, a measure that assesses visual abilities through tasks such as block design, even after controlling for the g-factor (Dolan et al. 2006; van der Sluis et al. 2006). One of the major strengths of the MGCFA approach is its suitability for analyzing sex/gender differences in intelligence, as it is considered the most appropriate method for such assessments (Gustafsson 1992; Molenaar et al. 2009). However, studies implementing MGCFA are often constrained by their reliance on standardization samples, which, while large, may not be sufficiently powered to detect subtle differences (Molenaar et al. 2009). Consequently, further research using MGCFA on adequately large and demographically representative samples is essential to definitively determine the role of the g-factor in sex differences within intelligence test scores (Molenaar et al. 2009).

While the current paper offers some insightful observation, it is important to acknowledge some limitations. For a start, the findings are derived from the Italian standardization of the Leiter-3, potentially impacting the generalizability of results to other cultures. A comparative analysis with results from other standardization samples could provide a more comprehensive understanding of cultural variations. The sample size of the Italian standardization, although designed to be representative of the Italian population, may not be sufficiently large to fully capture population diversity. This limitation could affect the statistical power of the results and should be considered when interpreting the findings. Moreover, the study did not thoroughly assess the impact of environmental factors, such as poverty or economic status, which might influence cognitive abilities. Future research should consider these factors for a more nuanced exploration of the topic. Additionally, conducting further analyses on a larger sample would allow for the exploration of whether sex/gender differences in performance are influenced by factors other than sex/gender, such as age or education. Incorporating these variables into the analysis could provide a more comprehensive understanding of the dynamics at play. Finally, qualitative methods, such as interviews, could offer a deeper exploration of the social and cultural contexts influencing sex/gender differences.

In future research, it is crucial to address the limitations highlighted and consider additional factors that could influence cognitive abilities, thereby contributing to a more robust and comprehensive understanding of sex/gender differences in intelligence. One notable consideration is the calculation of factors in the Leiter-3, where factors are typically derived from only two indicators, as seen in the memory and attention and inhibition batteries. This might pose challenges, particularly when employing MGCFA. An interesting avenue for future research could involve adopting a multi-battery approach, as proposed by Flanagan et al. (2007). Under these premises, several different batteries tapping intelligence could be used together on the same sample. In fact, as mentioned in the introduction the Leiter-3 is a language-free battery, reducing the impact of linguistic factors. However, future studies might benefit from including assessment methods allowing to assess the impact of verbal factors as well as the nonverbal ones. This approach will enable a more comprehensive assessment of the profile, incorporating tasks from different batteries within the same sample and under a unified theoretical framework, namely the CHC (Cattell-Horn-Carroll) approach. Alternatively, future meta-analysis could be implemented, not limited to a single battery but using several different batteries and components with the aim of providing a better understanding of sex/gender differences.

Our findings provide a nuanced contribution to the body of literature on sex/gender differences in intelligence, particularly by utilizing the Leiter-3 nonverbal intelligence battery within a MGCFA framework. Consistent with findings from Giofrè et al. (2022a, b) and studies employing the Wechsler scales, our research confirms the absence of differences in general intelligence (g-factor) across sexes using a MGCFA approach (see also Dolan et al. 2006; van der Sluis et al. 2006). However, it also underscores the presence of nuanced differences in specific cognitive domains, revealing that these subtleties extend beyond general cognitive ability and manifest in distinct areas of strength and weakness for each sex. These differences were favoring males in tasks requiring spatial manipulation and females in tasks demanding attention and inhibition control (Geary et al. 2021). This supports theories proposing that while general cognitive ability may not differ significantly between sexes, specific cognitive abilities can exhibit differences favoring both males and females (Johnson and Bouchard 2007). Additionally, Mackintosh (2011, p. 199) notes that while the two sexes do not differ significantly in average IQ, they do exhibit pronounced differences in components of IQ, particularly in spatial abilities. Our general conclusions are in line with those of Hunt (2011, p.406), who suggests that both biological and social influences contribute to the observable differences between men and women in cognitive abilities. Hunt argues that while there are predispositions that might lead to sex/gender cognitive trends, these are not definitive; social contexts and personal learning experiences can significantly modulate these predispositions. Therefore, our understanding of intelligence must appreciate the nuances in cognitive strengths and limitations across sexes, emphasizing that the question of whether men are more intelligent than women is not just unanswerable—it is the wrong question to ask. Our research thus extends the existing theories by providing empirical support from a nonverbal testing framework, which is less studied in the literature but critical for understanding cognitive abilities devoid of linguistic processing influences. These insights emphasize the importance of using diverse methodological approaches and testing batteries to fully capture the complexity of intelligence across genders, encouraging future studies to explore these subtle differences further with adequately powered samples.

Our findings also have significant clinical and educational implications. For example, assessments that heavily rely on visual or spatial rotation tasks may inadvertently favor male participants due to their generally stronger performance in these areas. Conversely, tasks that require sustained attention and the performance of routine activities over extended periods might provide an advantage to female participants. In educational settings, it can be argued that problem-solving exercises that depend heavily on spatial abilities or require manipulation of materials could unintentionally benefit males. Meanwhile, females, who typically exhibit better verbal comprehension and the ability to inhibit irrelevant information more efficiently, may find an advantage in tasks that are lengthy and less engaging. These observations suggest that sex/gender biases in task design and assessment criteria can significantly influence performance outcomes. Recognizing these nuances is crucial for developing more balanced and fair assessment practices in both clinical and educational contexts, ensuring that they do not unintentionally favor one sex/gender over the other. This awareness should inform the development of assessments and interventions that are sensitive to the diverse cognitive profiles of all students and clients.

To sum up, the current report offers significant insights into differences between males and females in general cognitive abilities. Our results suggest that, overall, males and females do not significantly differ in their general cognitive capacity (i.e., the g-factor). However, the study highlights the presence of specific strengths and weaknesses in certain factors and subtests. Our study opens avenues for future research. The identified strengths and weaknesses prompt intriguing questions that could deepen our understanding of the underlying mechanisms and factors contributing to sex/gender differences in cognitive abilities. This, we believe, contributes to the ongoing advancement of cognitive psychology as a field. This nuanced understanding is crucial for a more comprehensive and accurate portrayal of the cognitive profile of both males and females.

## Data Availability

The participants of this study did not give written consent for their data to be shared publicly, so due to the sensitive nature of the research, supporting data is not available.
